# Tetanus Vaccination and Extra-Immunization among Adult Populations: Eight-Year Follow Up Cohort Study of 771,443 Adults in Taiwan, 2006–2013

**DOI:** 10.3390/ijerph15081622

**Published:** 2018-08-01

**Authors:** Shih-Wei Liu, Liang-Chung Huang, Wu-Fu Chung, Jauching Wu, Li-Fu Chen, Yu-Chun Chen

**Affiliations:** 1 Department of Emergency Medicine, National Yang-Ming University Hospital, I-Lan 26042, Taiwan; shihweiliu123@gmail.com (S-W.L.); horus7855@yahoo.com.tw (L-C.H.); wolfchung2001@yahoo.com.tw (W-F.C.); 2 School of Medicine, National Yang-Ming University, Taipei 11221, Taiwan; jauching@gmail.com; 3 Department of Neurosurgery, Neurological Institute, Taipei Veterans General Hospital, Taipei 11217, Taiwan; 4 Institute of Hospital and Health Care Administration, National Yang-Ming University, Taipei 11221, Taiwan; 5 Department of Family Medicine, Taipei Veterans General Hospital, Taipei 11217, Taiwan

**Keywords:** tetanus boosters, vaccination coverage, extra-immunization, cohort study

## Abstract

Under-and extra-immunization of tetanus boosters are important issues to consider in reducing the burden of vaccine-preventable disease in adults. The present study aimed to analyze the trend of vaccination coverage (VC) and risk factors associated with extra-immunization of tetanus during an 8-year period using a national-scale cohort database. Taiwan’s one-million representative research database, the Longitudinal Health Insurance Database (LHID2005) was used. A total of 771,443 adults aged between 20 and 79 years were enrolled and followed from 1 January 2006 to 31 December 2013. VC at the beginning was as low as 35.1%, declining gradually and dropping to 33.9% at the end of follow-up. While a total of 303,480 tetanus boosters were used during the study period, more than half (55.5%) of these boosters were considered as extra-immunized. Both individual characteristics and visit characteristics were strongly associated with extra-immunization. Males, young and older adults, and those with a higher number of comorbidities were more likely to receive extra-immunization boosters, especially when they had more severe symptoms, visited an emergency room, or visited a hospital with lower accreditation levels located in a less urbanized area. This information could enhance implementation of evidence-based programs for tetanus boosters.

## 1. Introduction

Tetanus-related death is a rare but increasing concern among adult population nowadays while recent surveys have shown that immunity to tetanus continues to wane among adults, making tetanus a continuing threat with increasing age in adults [1,2,3]. Routine vaccination with decennial tetanus boosters (DTB) was first recommended by the Advisory Committee on Immunization Practices (ACIP) for US adults in 2005, and more than 40 countries have already updated their recommendations on regular administration of tetanus boosters [4]. However, vaccination coverage (VC) against tetanus remains low across some nations (with VC varying from 40% to 60% per country) mainly because of low collective awareness of tetanus as well as a lack of provider-to-patient vaccination recommendations; thus, vaccine coverage data is crucial for improved coverage [5,6,7,8,9].

Extra-immunization against tetanus, that is, administering more than the required amount of regular booster or at a shortened interval regardless of previous vaccine status, has caused both safety and economic concerns even though continuous efforts have been made to improve vaccination coverage among adults [10]. Although it is undesirable and unnecessary, extra-immunization still occurs because of a lack of ready access to complete and accurate immunization records [11,12]. Redundant booster administration has been reported in approximately 57% of wounded patients in emergency departments because most adults were under-vaccinated and over half of patients had incorrect memory on their tetanus vaccination status. The default in wound treatment guides is to give tetanus boosters to any patient without a clear vaccination history [13,14]. While having a low threshold for tetanus boosters at the point of wound care may increase tetanus vaccination coverage, extra-immunization may increase the risk of unnecessary adverse effects and vaccine wastage costs that are borne by an already strained healthcare system [15,16,17,18]. There is high demand for a detailed vaccination strategy to balance under-immunization and extra-immunization against tetanus but this is limited by a paucity of epidemiologic information about the dynamic changes that take place through the whole vaccination delivery process.

The present study aimed to analyze the change in vaccination coverage (VC) and risk factors associated with extra-immunization of tetanus during an 8-year period using a national-scale cohort database. The National Health Insurance Research Database (NHIRD) of Taiwan, covering the entire population of 23 million insurants and accumulated since 1997, provided a unique opportunity to observe the long-term change in the immunization status of tetanus of the whole cohort [19]. This database features monopolistic coverage of more than 99% of the population, unrestricted access to healthcare providers of the patients’ choice, and comprehensive records of procedures. Therefore, this study allowed access to the almost complete history of patients before and after receiving tetanus vaccines over a 20-year span that covered the whole course of tetanus vaccination. To date, this is the largest sample with a long-time follow up used to investigate the natural history of the immunization status of tetanus—such unique information could be valuable and help to optimize immunization programs.

## 2. Materials and Methods

### 2.1. Data Source and Ethical Concerns

Since 1995 Taiwan’s Government has implemented a National Health Insurance (NHI) program, which provides unrestricted access to medical care and universal health insurance for all residents in Taiwan. The NHI program has comprehensively enrolled 99% of the Taiwanese population and contracted with 97% of the providers of healthcare services in Taiwan. The data was named NHIRD and has been released to the public for research purposes after a vigorous encryption de-identification and anonymization process. The NHIRD contains comprehensive information on the insured subjects, including gender, date of birth, dates of clinical visits (both preventive services and emergent visits), the International Classification of Diseases (Ninth Revision) Clinical Modification (ICD-9-CM) codes of diagnoses, details of prescriptions, expenditure amounts, and characteristics of health providers, etc.

This study was initiated after approval from the Institutional Review Board of the National Yang-Ming University Hospital, Taiwan (NYMUH IRB No. 2014A020) and the National Yang-Ming University, Taiwan. The Institutional Review Board waived the requirement for written informed consent from each of the patients involved since all identifying personal information in NHIRD is encrypted.

### 2.2. Identification of Study Cohort

This was a population-based retrospective cohort study. A representative set of NHIRD called the Longitudinal Health Insurance Database (LHID) was used as the study cohort. The LHID2005 contains one million people who were sampled from the whole population (25.68 million individuals) registered in Taiwan at the end of 2005. The LHID2005 comprises anonymous, comprehensive and representative data on the health care of the entire Taiwan population and the medical history of each enrolled individual can be traced back to 1995, which provides an extraordinarily comprehensive immunization history over at least ten years.

We enrolled all adults aged between 20 and 79 years on 1 January 2006, when follow up of the cohort was initiated. For each selected individual, we extracted his/her history of tetanus vaccination (number of doses reimbursed and age at delivery) and followed up until 31 December 2013, the end of the study, or the date out-of-cohort (death or emigration) depending on which came first. We were also able to trace each subject’s prior vaccination history for up to 10 years before enrollment (1 January 1996, at the earliest) using LHID2005.

### 2.3. Ascertaining Immunity Status Against Tetanus

In the current study, we defined tetanus boosters as inclusive of all tetanus toxoid-containing vaccines, including tetanus toxoid (TT) vaccine, tetanus and diphtheria (Td) vaccine and tetanus, diphtheria, and pertussis (Tdap) vaccine administered to recipients aged 20 years and over. Annual administration rates of tetanus boosters were calculated by dividing the total doses of administered tetanus boosters by the number of the target population.

The participants were classified into either of the following groups according to their immunity status against tetanus: “live with effective immunity’” and “live without effective immunity”. We assumed that participants gained effective tetanus immunity, (that is they became live with effective immunity), immediately following the date they received a tetanus booster. According to previous large serology studies, we also assumed that immunity waned after ten years equally for every tetanus booster recipients [20,21]. As a result, every participant who lives with effective tetanus immunity would become live without effective immunity unless another booster was given according to the schedule (a latency window of 3 months was allowed). Participants were marked with “death” if the date of death came before the end of follow up.

### 2.4. Estimation of Vaccination Coverage (VC) and Extra-Immunization

Vaccination coverage (VC) was calculated by dividing the number of adults live with effective tetanus immunity by the number of the target population and 95% confidence intervals were also estimated.

Tetanus boosters were categorized as either an effective booster or an extra-immunized booster, according to the recipient’s tetanus immunity. A tetanus booster was considered extra-immunized if the recipient already had effective tetanus immunity from a recent booster within the recommended interval (10 years with a 3-month overlap). 

### 2.5. Characteristics of Visits for Tetanus Boosters

To further assess risk factors associated with extra-immunization, recipients’ demographics as well as characteristics of visits for tetanus boosters were further analyzed. Subjects’ co-morbidities were categorized according to Elixhauser’s co-morbidity model by the presence of either diagnostic codes in the outpatient records or discharge codes in the database within two years before the date of the visit [22]. The severity of the subject’s condition was determined according to levels of triage and classified into four levels including immediate resuscitation, very urgent, urgent and non-urgent, whereas the severities of visits in out-patient-department were assumed to be non-urgent. The characteristics of visits, including place of visit, accreditation level, urbanization level and geolocation of the hospital were extracted from the database.

### 2.6. Statistical Analysis

All of the data were linked using the SQL server 2017 (Microsoft Corp, Redmond, WA, USA) and analyzed by Stata software (Statacorp, College Station, TX, USA). The annual administration rate of tetanus boosters and VC were calculated to demonstrate the long-term change of VC in the study cohort. A logistic regression model was used to assess risk factors associated with extra-immunization. Adjusted odds ratio (AOR) for extra-immunization for each factor was estimated by controlling other factors in the model. The predicted probability of receiving extra-immunized tetanus boosters was calculated for individuals of each characteristic as they were average on all other characteristics. The base probability was calculated for individuals who were average on all characteristics. The probability of extra-immunization and the difference from the base probability of extra-immunization, both expressed in percentage, were calculated to quantify the effect of levels of each factor. A two-tailed level of 0.05 was considered statistically significant.

## 3. Results

### 3.1. Annual Administration Rates, Extra-Immunization Rates, and Vaccination Coverage

The study cohort enrolled 771,443 adults aged between 20 and 79 years and the entire cohort was followed up from 1 January 2006 to 31 December 2013. Participants were completely observed throughout the study period with a minimal loss (lost follow-up rate = 0.02%, n = 184).

**Figure 1:**
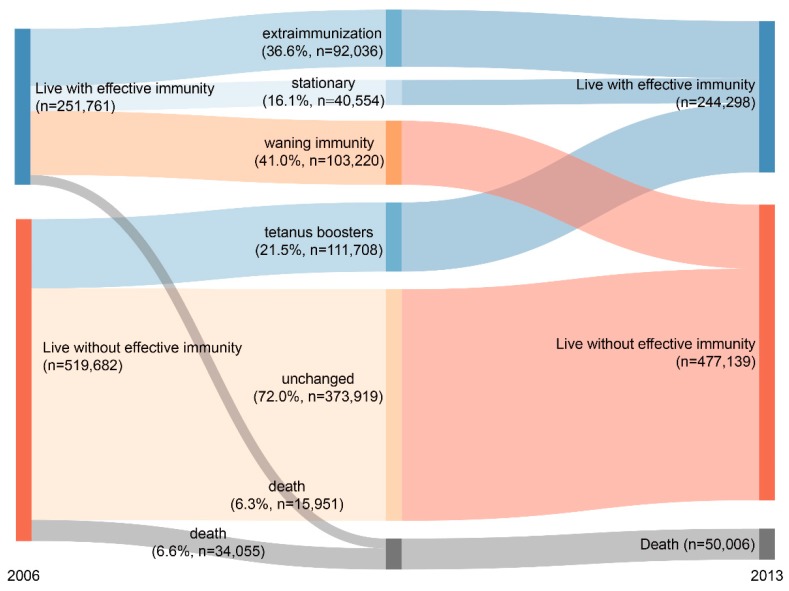
Interval change of immunity status against tetanus among adults aged between 20 and 79 years in Taiwan before and after 8-year follow up. (2006–2013; n = 771,443).

Figure 1 demonstrates the interval changes in immunity against tetanus for the entire study cohort before and after the 8-year follow up. At the beginning of the study, barely one-third of adults ha effective immunity against tetanus, the VC was 35.1% (245,016 participants had effective immunity vs. 526,427 participants who did not). After eight years, nearly half (41.0%, n = 111,708, 14.5% of entire cohort) of participants’ immunity waned whereas only a portion (21.5%, n = 103,220, 13.2% of entire cohort) of participants received tetanus boosters. Furthermore, quite a significant portion of participants (36.6%) were extra-immunized with excessive tetanus boosters. All the above changes led to an overall decline in VC of the entire cohort (Figure 1, Figure S1).

During the 8-year follow up, the entire cohort received a total of 303,480 doses of tetanus booster with an administrative rate at 39.3%. Only one out of twenty adults received a tetanus booster every year, the annual administration rates decreased gradually from 5.4% to 4.8%. Moreover, more than half (55.5%, 168,413) of these boosters were considered as extra-immunized, which are excessively administered to recipients who already have effective tetanus immunity from recent boosters within the past ten years. As a result, the VC declined slightly with aging of the entire cohort and it finally dropped to 33.9% at the end of follow up, 1.2% lower than the beginning of the study (Table 1).

### 3.2. Change in Age-Specific Vaccination Coverage

**Figure 2:**
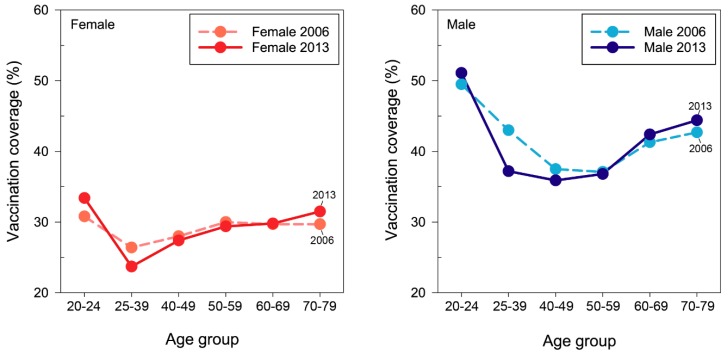
Tetanus vaccination coverage among adults aged between 20 and 79 years in Taiwan’s representative cohort at the beginning and the end of 8-year follow up by age and gender. (2006–2013, n = 771,443).

Figure 2 suggests a substantial difference in distributions of VC across gender and age groups. The age distribution of VC was roughly U-shaped, reflecting the dominance of effective immunity in young and older adults than in middle-aged adults. VC was highest in the 20s, but dropped sharply to the lowest in those aged 25–39 years, and then increased gradually with age after the 40s. Moreover, males clearly had a higher rate of effective immunity than females; the VC of females was invariably about 25% lower than that of males in every age group (Figure 2).

Table 2 shows that young and older adults were more likely to receive tetanus boosters than middle-age adults since there is a considerable age difference in change in VC through the eight-year follow up. VC showed the highest increase in the young adult group (the twenties) followed by the eldest group (70–79 years) and older group (60–69 years) at the end of the study for both females and males. The findings shown in Figure 2 and Table 2 may reflect an age-gender dependent gap for tetanus boosters for adults.

### 3.3. Risk Factors for Extra-Immunization Boosters

To further assess the risk factors for extra-immunization boosters, we analyzed 303,480 visits for tetanus boosters made by 214,847 recipients during the observation period. Extra-immunization boosters represented a considerable portion of total visits (55.5%; 168,413 visits out of 303,480 visits were for extra-immunization boosters). Half of the recipients (49.9%, 107,227 adults) were extra-immunized with only a few recipients administered with more than 2 boosters during the study period. A logistic regression model showed that both individual characteristics and visit characteristics were significantly associated with extra-immunization boosters; males, young and older adults, and those with an increased number of comorbidities were more likely to receive extra-immunization boosters especially when they had more severe symptoms, visited an emergency room, or visited a hospital with lower accreditation levels located in a less urbanized area (Table 3).

**Figure 3:**
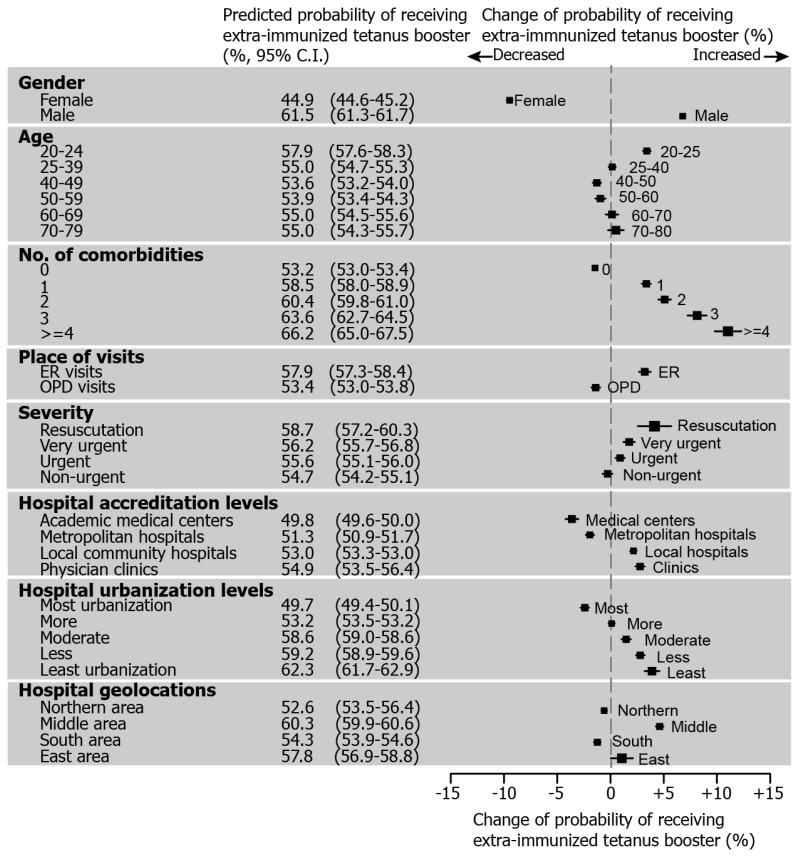
Predicted probability of receiving extra-immunized tetanus boosters and change from base probability by recipient demographics and visit characteristics among tetanus booster recipients aged between 20 and 79 years in Taiwan. (2006–2013; recipient number, 214,847; visit number, 303,480).

Figure 3 quantifies and illustrates the effect of each level of risk factors on the probability of extra-immunization, where the most influential factor for extra-immunization was gender followed by the number of comorbidities and hospital urbanization level (the probability of extra-immunization included: females 44.9% vs. males 66.5%; zero comorbidities 53.2% vs. ≥ 4 comorbidities 63.2%; most urbanization 49.7% vs. least urbanization 62.3%). Males were more likely to receive extra-immunization boosters, while the age distribution of the probability of extra-immunization was roughly U-shaped. The likelihood of extra-immunization also increased with increasing number of comorbidities. 

There is a tendency for extra-immunization boosters to be frequently used in urgent situations. The emergency room was the place most prone to give extra-immunization compared to out-patient-departments (the probability of extra-immunization for an ER visit was 57.9% vs. 53.4% for an OPD visit). Moreover, the probability of extra-immunization during the most urgent visits was 4% higher than non-urgent visits (immediate resuscitation visits were 58.7% vs. 54.7% for non-urgent visits) (Figure 3).

It appears that hospital-related factors are linked to extra-immunization. Visits to academic medical centers and metropolitan hospitals are less likely to result in extra-immunization compared to local hospital and clinics. The degree of urbanization of the location of the hospital may decrease the probability of extra-immunization (most urbanization 49.7% vs. least urbanization 62.3%) (Figure 3).

## 4. Discussion

Both under-immunization and extra-immunization are a continuing threat to adults and the preventive healthcare system. While one’s effective immunity against tetanus usually wanes every ten years if there is no routine decennial tetanus boosters, one could also easily be extra-immunized because of the lack of an accurate immunization history. Epidemiologic information from large-scale observational studies is needed. The current study uniquely followed the dynamic change in effective immunity against tetanus (in terms of VC) of 771,000 adults aged between 20 and 79 years for eight years. We found that the effective immunity of the entire cohort degraded gradually despite the ready use of tetanus boosters at the point of wound care. Moreover, a large portion of tetanus boosters were extra-immunized. Both individual characteristics and visit characteristics were strongly associated with extra-immunization.

Our study provided convincing support for detailed tetanus booster programs for adults. Relying solely on tetanus boosters at the point-of-care is not sufficient to counter waning tetanus immunity. In our study on the change in VC, we showed that even though Taiwan has readily accessible and affordable medical services, their contribution to protection against tetanus is still minimal; furthermore, more than half of the administered boosters were extra-immunized. Therefore, we suggest that future immunization strategies should be stratified by age and gender. The current study showed a clear age and gender gap in the administration of tetanus boosters. This discrepancy may be explained by patients’ misconceptions about immunization recommendations as well as attitudes towards vaccinations [5]. Therefore, a detailed tetanus booster program stratified by age and gender, with specific information disseminated through media campaigns might be helpful to increase vaccination coverage.

Understanding the factors that contribute to extra-immunization is crucial in reducing its incidence. To our knowledge, this is the first study to investigate the occurrence and factors for extra-immunization using a large and comprehensive database. It had been reported that there are higher rates of extra-immunization in vulnerable and at-risk populations in the US, including those in underprivileged racial/ethnic minority groups [11]. In the multiple logistic regression model, we found both individual and provider characteristics were strongly associated with extra-immunization. More specifically, recipients who were males, young and older adults, or who had a higher number of comorbidities were more likely to receive extra-immunization boosters, especially when they had more severe symptoms, visited an emergency room, or visited a hospital with lower accreditation levels located in a less urbanized area.

Centralized immunization registries could facilitate tetanus immunization for adults. Immunization history from patients can often be misleading because of incorrect memory [17]. In Taiwan, most young males receive tetanus boosters during compulsory military service while young females are likely to receive tetanus boosters when pregnant, which might contribute to the peak in extra-immunization rate in young adults. Darden et. al. suggested that extra-immunization could be a result of a fragmented health care system because of poor connection among health care givers-to-health care givers and health care givers-to-adult populations [11]. Moreover, lack of readily accessible immunization histories may make providers located in rural areas and local clinics more prone to extra-vaccination. A database would keep track of individuals’ immunization histories and prevent providers from extra-vaccination by determining when shots are due [23].

A claim or reimbursement database, could be an effective alternative to resource-consuming surveys that are commonly used in traditional immunization programs. Our study represents a novel approach for reusing routinely collected data. Since it is impossible to measure serum protective tetanus antitoxin level for the whole population, by assuming the efficacy of tetanus according to previous seroepidemiology studies, we were able to closely observe the vaccination coverage trends through the use of vaccine reimbursement data. This new method represents an innovative approach to provide timely, updated, reliable immunization information that enables program managers to do real-time monitoring, investigate potential problems and take appropriate remedial action to improve performance.

Our results provide compelling evidence for long-term observation of the trends in tetanus effective immunity of an entire cohort. Additionally, our study offers an understanding of incidence and risk factors for extra-immunization. Both would enhance implementation of evidence-based programs for tetanus boosters. However, some limitations are worth noting. First, the current study assumed that effective immunity against tetanus wanes after ten years equally for all particpants in the cohort. This would underestimate vaccination coverage but overestimate extra-immunization in young adults and middle-aged adults. A more sophisticated mathematical model should be considered for accurate estimation. Second, detailed clinical notes are not available in our database. Thus, in the present study we were not able to determine the appropriateness of tetanus boosters for poor titers, immuno-compromised patients or severe wounds. As a result, extra-immunization could be slightly overestimated. Third, some persons may purchase their vaccine without claiming reimbursement. In Taiwan’s national health insurance scheme, everyone has equal and full access to wound care as well as to vaccination services. Thus, this issue is minimized in the current study. Fourth, errors/duplications in record keeping (particularly in less advanced, less urbanized clinics) involving vaccination dates or doses may underestimate extra-immunization rates. A field study of immunization status among such minority/underprivileged groups is warranted.

## 5. Conclusions

The current study provides compelling evidence about the trend of effective immunity and risk factors for extra-immunization by following a representative cohort. Vaccination coverage declines gradually without a universal immunization program. A high percentage of extra-immunization tetanus boosters were noted. Both individual characteristics and visit characteristics were strongly associated with extra-immunization. This information would enhance the implementation of evidence-based programs for tetanus boosters.

## Figures and Tables

**Table 1:** Annual trends of tetanus administration rates, proportion of extra-immunization boosters and vaccination coverage among adults aged between 20 and 79 years in Taiwan’s representative cohort. (2006–2013, n = 771,443)

Year	# of Cohort	Tetanus Boosters		Extra-Immunized Tetanus Boosters ^2^		Vaccination Coverage (VC, %) ^3^
		# of Cose	Annual Administration Rate (%) ^1^	# of Doses	% of Boosters	
2006	771,443	41,913	5.4	23,072	55.0	35.1
2007	763,951	40,598	5.3	22,793	56.1	35.4
2008	757,278	39,014	5.2	21,624	55.4	35.3
2009	750,850	38,238	5.1	21,309	55.7	34.9
2010	745,270	37,712	5.1	21,095	55.9	34.7
2011	739,592	35,971	4.9	20,021	55.7	34.4
2012	733,353	35,365	4.8	19,681	55.7	34.1
2013	727,048	34,669	4.8	18,818	54.3	33.9
Total		303,480		168,413		

^1^ Annual administration rates were calculated by dividing the total doses of administered tetanus boosters by the number of the target population. ^2^ Tetanus boosters were considered extra-immunized if the recipient already had effective tetanus immunity by a recent booster within 10 years. ^3^ Vaccination coverage (VC) was calculated by dividing the number of recipients who had effective tetanus immunity by the number of target population.

**Table 2:** Tetanus vaccination coverage (VC, %) and percentage change among adults aged between 20 and 79 years in Taiwan’s representative cohort at the beginning and end of 8-year follow up by age and gender (2006–2013, n = 771,443).

Age	VC at the Beginning (1st Year, 2006) of Follow Up (%)			VC at the End (8th Year, 2013) of Follow Up (%)			% Change of VC		
	Female	Male	Total	Female	Male	Total	Female	Male	Total
20–24	30.8	49.5	39.6	33.4	51.1	41.7	8.6	3.2	5.4
25–39	26.4	43.0	34.5	23.7	37.2	30.2	−10.4	−13.6	−12.4
40–49	28.0	37.5	32.8	27.4	35.9	31.6	−2.1	−4.5	−3.6
50–59	30.0	37.1	33.5	29.4	36.8	33.0	−2.0	−1.0	−1.7
60–69	29.7	41.3	35.2	29.8	42.4	35.5	0.3	2.7	0.9
70–79	29.7	42.7	36.4	31.5	44.4	37.7	6.3	4.0	3.6
All ages	28.5	42.1	35.1	28.0	40.2	33.9	−1.8	−4.5	−3.6

**Table 3:** Characteristics of visits for tetanus boosters and adjusted odds ratios for extra-immunized tetanus boosters among tetanus booster recipients aged between 20 and 79 years in Taiwan. (2006–2013; recipient number, 214,847; visit number, 303,480).

	Visits for Tetanus Boosters n = 303,480 # of Visits, (% of Visits)		Adjusted Odds Ratio (AOR) for Extra-Immunization Boosters AOR, (95% C.I.)		*p*-Value Sig. ^1^
**Individual characteristics**					
**Gender**					
Male	186,684	(61.5)	1.96	(1.93–1.99)	<0.001 ***
Female	116,796	(38.5)	(ref)		
**Age**					
20–24	71,261	(23.5)	1.22	(1.19–1.25)	<0.001 ***
25–39	85,663	(28.2)	1.06	(1.04–1.09)	<0.001 ***
40–49	58,002	(19.1)	(ref)		
50–59	41,911	(13.8)	1.01	(0.99–1.04)	0.28
60–69	28,062	(9.2)	1.06	(1.03–1.09)	<0.001 ***
70–79	18,581	(6.1)	1.08	(1.40–1.12)	<0.001 ***
**Number of comorbidities**					
0	208,814	(68.8)	(ref)		
1	50,918	(16.8)	1.23	(1.21–1.26)	<0.001 ***
2	26,906	(8.9)	1.32	(1.30–1.38)	<0.001 ***
3	11,264	(3.7)	1.51	(1.47–1.60)	<0.001 ***
≥4	5578	(1.8)	1.72	(1.63–1.82)	<0.001 ***
**Visit characteristics**					
**Place of visit**					
Emergency room	177,054	(58.3)	1.22	(1.17–1.26)	<0.001 ***
Out-patient department	126,426	(41.7)	(ref)		
**Severity**					
Immediate resuscitation	3707	(1.2)	1.21	(1.12–1.30)	<0.001 ***
Very urgent	47,177	(15.5)	1.09	(1.05–1.13)	<0.001 ***
Urgent	110,342	(36.4)	1.05	(1.02–1.09)	0.004 **
Non-urgent	142,254	(46.9)	(ref)		
**Hospital accreditation levels**					
Academic medical centers	33,093	(10.9)	(ref)		
Metropolitan hospitals	85,806	(28.3)	1.07	(1.04–1.10)	<0.001 ***
Local community hospitals	98,814	(32.6)	1.27	(1.24–1.31)	<0.001 ***
Physician clinics	85,767	(28.3)	1.31	(1.26–1.35)	<0.001 ***
**Hospital urbanization levels**					
Most urbanization	67,889	(22.4)	(ref)		
More	106,891	(35.2)	1.11	(1.09–1.13)	<0.001 ***
Moderate	49,643	(16.4)	1.18	(1.15–1.21)	<0.001 ***
Less	58,073	(19.1)	1.25	(1.21–1.28)	<0.001 ***
Least urbanization	20,984	(6.9)	1.31	(1.26–1.35)	<0.001 ***
**Hospital geolocations**					
Northern area	131,803	(43.4)	1.03	(1.01–1.05)	0.004 **
Middle area	73,905	(24.4)	1.28	(1.26–1.31)	<0.001 ***
South area	89,188	(29.4)	(ref)		
East area	8584	(2.8)	1.10	(1.05–1.15)	<0.001 ***

^1^ Statistical significance, **: *p* < 0.01, ***: *p* < 0.001

## Supplementary Materials

The following are available online at http://www.mdpi.com/1660-4601/15/8/1622/s1, Figure S1: Interval change of immunity status against tetanus among adults aged between 20 and 79 years in Taiwan before and after 8-year-follow-up. (2006–2013; n = 771,443)
