# Supplementary material for: Tetanus Vaccination and Extra-Immunization among Adult Populations: Eight-Year Follow Up Cohort Study of 771,443 Adults in Taiwan, 2006–2013
Source: Int J Environ Res Public Health. 2018 Aug 1;15(8):1622. doi: 10.3390/ijerph15081622 (PMC6121571; doi:10.3390/ijerph15081622)
Supplement: Supplementary file 1 [file ijerph-15-01622-s001.pdf]

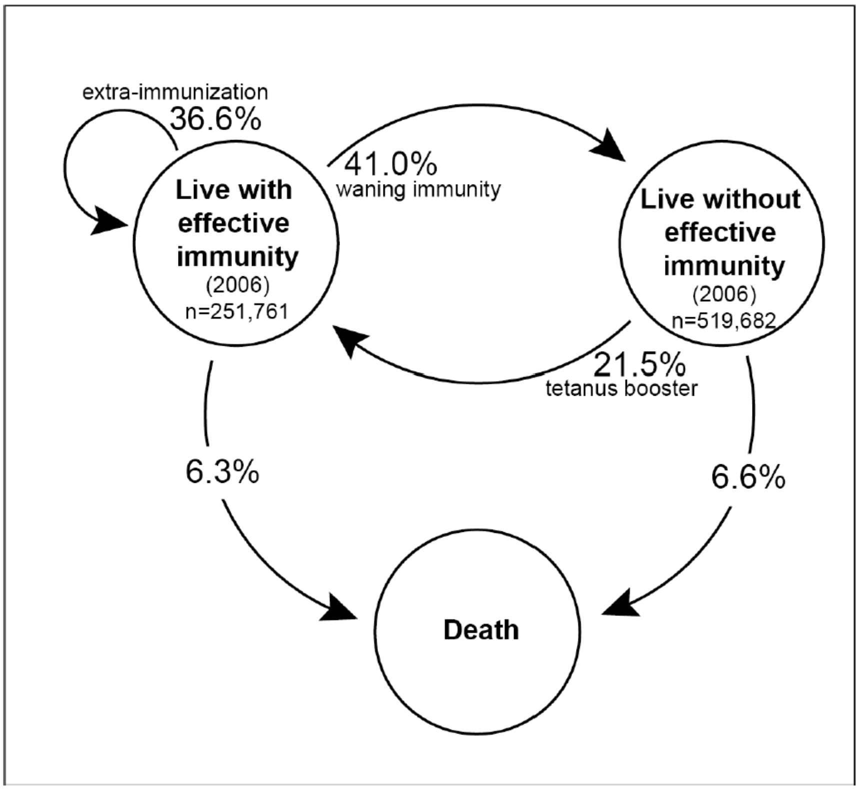

Figure S1. Interval change of immunity status against tetanus among adults aged between 20 and 79 years in Taiwan before and after 8-year-follow-up. (2006–2013; n = 771,443)
